# DEMOGRAPHICS’ IMPACT ON CURRENT AND NOVEL NEUROPSYCHOLOGICAL MEASURES FOR ALZHEIMER’S DISEASE SCREENING

**DOI:** 10.1093/geroni/igae098.2585

**Published:** 2024-12-31

**Authors:** Alexandra Reed, Kevin Duff, Sydney Schaefer

**Affiliations:** Arizona State University, Phoenix, Arizona, United States; Oregon Health & Science University, Portland, Oregon, United States; Arizona State University, Tempe, Arizona, United States

## Abstract

Neuropsychological assessments are often affected by demographic factors, such as age, education, and sex. These demographic variables frequently lead to differences in test scores between minorities and non-minorities, lowering diagnostic accuracy of Alzheimer’s disease (AD) among minoritized groups. A new test (quick Behavioral Exam to Advance Neuropsychological Screening, or qBEANS) has been developed to assess early signs of AD. This study compared the influence of demographic variables on qBEANS and other neuropsychological tests that are commonly used in the assessment of AD. In this study, 165 participants (68 cognitively unimpaired; 52 amnestic Mild Cognitive Impairment; 45 mild AD) completed the Symbol Digit Modalities Test (SDMT), Hopkins Verbal Learning Test-Revised (HVLTR); Brief Visuospatial Memory Test-Revised (BVMTR); and Trails Making Test (TMT) A and B. Participants also completed the qBEANS, which involves spooning two raw kidney beans at once with the nondominant hand to one of three target cups in a simple sequence, with higher scores being worse performance. Results indicated that significant age effects were observed for all tests except qBEANS and TMT-A, with older ages being associated with worse scores. Similarly, there were significant education effects for all tests except qBEANS and TMTs A and B. Only the HVLTR (total and delayed recall) showed a significant sex effect, with females performing better than males. These data suggest that qBEANS may be appropriate for screening diverse populations without controlling for demographic variables.

